# Supplementary material for: Breast Cancer Care Pathways for Women with Preexisting Severe Mental Disorders: Evidence of Disparities in France?
Source: J Clin Med. 2023 Jan 4;12(2):412. doi: 10.3390/jcm12020412 (PMC9862837; doi:10.3390/jcm12020412)
Supplement: Supplementary file 1 [file jcm-12-00412-s001.zip › jcm-2084980-supplementary.pdf]

Figure S1. Flowchart for the selection of the study population

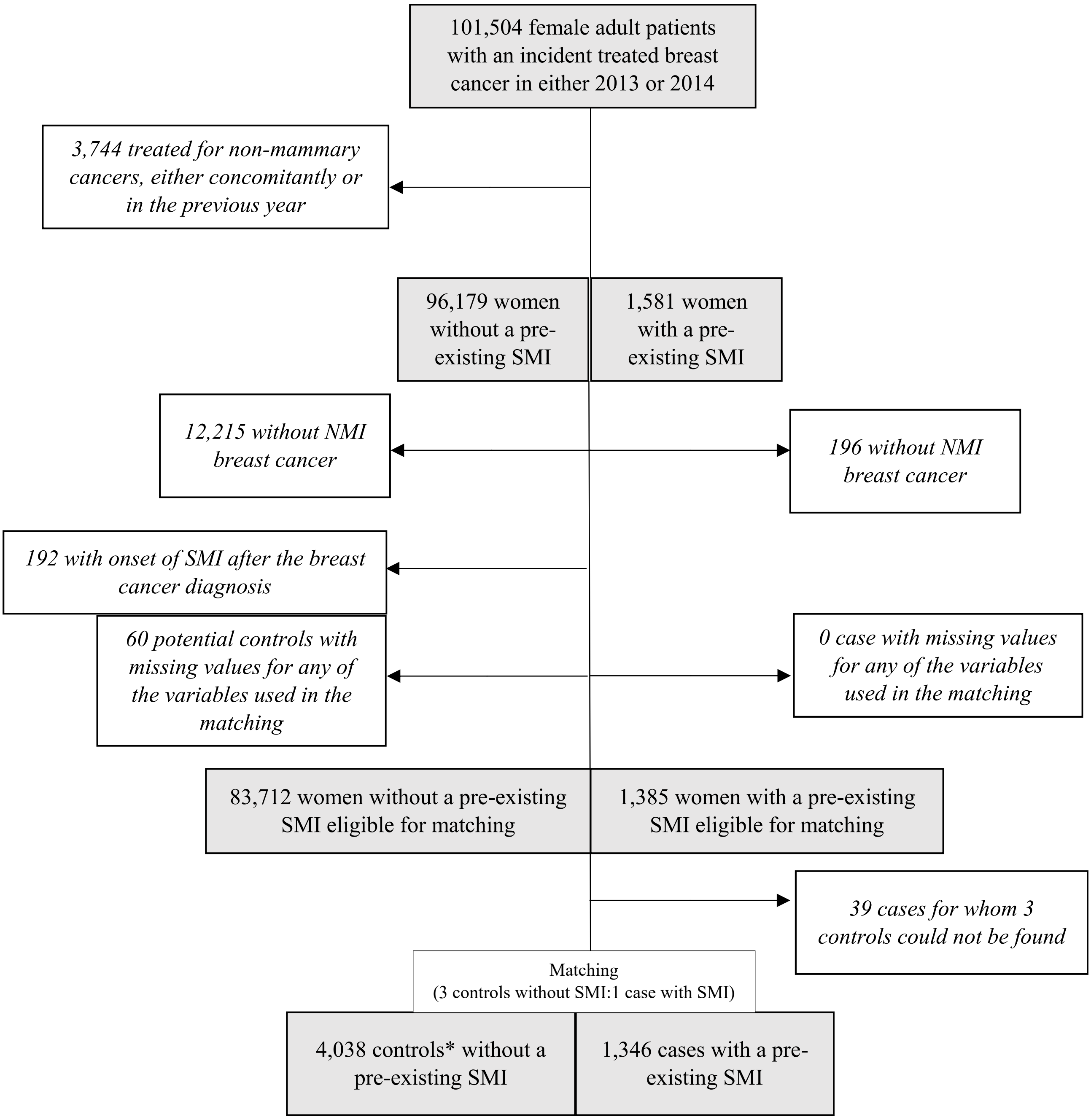

Note: *4,000 individuals selected through matching with replacement (certain matched multiple times to obtain 4,038 controls)

Table S1. Characteristics of matched and unmatched SMI patients

	Matched women with SMI (n=1,346)	Unmatched women with SMI (n=39)
**Characteristic**	**Mean (±SD) or n (%)**	
**Demographics**		
Age	61.08 (±11.70)	53.82 (±18.14)
**Socio-economic characteristics at the individual level**		
Inclusion in the scheme covering healthcare costs for low-income groups (CMU-C)	52 (3.86)	5 (12.82)
Inclusion in the scheme providing financial assistance for the purchase of supplementary health insurance (ACS)	155 (11.52)	6 (15.38)
**Characteristics of the living environment**		
Quintile of community-level deprivation index (FDep)– using bounds calculated at the national level		
1st quintile (least deprived)	249 (18.50)	1 (2.56)
2nd quintile	229 (17.01)	4 (10.26)
3rd quintile	333 (24.74)	10 (25.64)
4th quintile	263 (19.54)	12 (30.77)
5th quintile (most deprived)	228 (16.94)	9 (23.08)
Missing values	44 (3.27)	2 (5.13)
Residency in an overseas territory	23 (1.71)	2 (5.13)
**Clinical characteristics**		
Non-metastatic invasive breast cancer with lymph node involvement	263 (19.54)	30 (76.92)
Non-metastatic invasive breast cancer with no lymph node involvement	1,083 (80.46)	8 (20.51)
Comorbidity index	0.58 (±0.97)	0.37 (±0.67)
